# Validation of an automatic tool for the rapid measurement of brain atrophy and white matter hyperintensity: QyScore®

**DOI:** 10.1007/s00330-021-08385-9

**Published:** 2022-01-01

**Authors:** Enrica Cavedo, Philippe Tran, Urielle Thoprakarn, Jean-Baptiste Martini, Antoine Movschin, Christine Delmaire, Florent Gariel, Damien Heidelberg, Nadya Pyatigorskaya, Sébastian Ströer, Pierre Krolak-Salmon, Francois Cotton, Clarisse Longo dos Santos, Didier Dormont

**Affiliations:** 1 Qynapse SAS, 130 rue de Lourmel, 75015 Paris, France; 2 Equipe-Projet ARAMIS, ICM, CNRS UMR 7225, Inserm U1117, Sorbonne Université UMR_S 1127, Centre Inria de Paris, Groupe Hospitalier Pitié-Salpêtrière Charles Foix, Faculté de Médecine Sorbonne Université, Paris, France; 3 Fondation Adolphe de Rothschild, Paris, France; 4 Department of Neuroradiology, University Hospital of Bordeaux, Bordeaux, France; 5 Faculty of Medicine, Claude-Bernard Lyon 1 University, 69000 Lyon, France; 6 Service de Radiologie and Laboratoire d’anatomie de Rockefeller, centre hospitalier Lyon Sud, hospices civils de Lyon, 69000 Lyon, France; 7 Department of Neuroradiology, Groupe Hospitalier Pitié-Salpêtrière, AP-HP, Sorbonne Université UMR_S 1127, Paris, France; 8 Clinical and Research Memory Centre of Lyon, Hospices Civils de Lyon, Lyon, France; 9 University of Lyon, Lyon, France; 10 INSERM, U1028; UMR CNRS 5292, Lyon Neuroscience Research Center, Lyon, France; 11 Radiology Department, centre hospitalier Lyon-Sud, hospices civils de Lyon, 69310 Pierre-Bénite, France; 12 Inserm U1044, CNRS UMR 5220, CREATIS, Université Lyon-1, 69100 Villeurbanne, France

**Keywords:** Magnetic resonance imaging, White matter, Hippocampus, Automated quantification

## Abstract

**Objectives:**

QyScore® is an imaging analysis tool certified in Europe (CE marked) and the US (FDA cleared) for the automatic volumetry of grey and white matter (GM and WM respectively), hippocampus (HP), amygdala (AM), and white matter hyperintensity (WMH). Here we compare QyScore® performances with the consensus of expert neuroradiologists.

**Methods:**

Dice similarity coefficient (DSC) and the relative volume difference (RVD) for GM, WM volumes were calculated on 50 3DT1 images. DSC and the F1 metrics were calculated for WMH on 130 3DT1 and FLAIR images. For each index, we identified thresholds of reliability based on current literature review results. We hypothesized that DSC/F1 scores obtained using QyScore® markers would be higher than the threshold. In contrast, RVD scores would be lower. Regression analysis and Bland–Altman plots were obtained to evaluate QyScore® performance in comparison to the consensus of three expert neuroradiologists.

**Results:**

The lower bound of the DSC/F1 confidence intervals was higher than the threshold for the GM, WM, HP, AM, and WMH, and the higher bounds of the RVD confidence interval were below the threshold for the WM, GM, HP, and AM. QyScore®, compared with the consensus of three expert neuroradiologists, provides reliable performance for the automatic segmentation of the GM and WM volumes, and HP and AM volumes, as well as WMH volumes.

**Conclusions:**

QyScore® represents a reliable medical device in comparison with the consensus of expert neuroradiologists. Therefore, QyScore® could be implemented in clinical trials and clinical routine to support the diagnosis and longitudinal monitoring of neurological diseases.

**Key Points:**

*• QyScore® provides reliable automatic segmentation of brain structures in comparison with the consensus of three expert neuroradiologists.*

*• QyScore® automatic segmentation could be performed on MRI images using different vendors and protocols of acquisition. In addition, the fast segmentation process saves time over manual and semi-automatic methods.*

*• QyScore® could be implemented in clinical trials and clinical routine to support the diagnosis and longitudinal monitoring of neurological diseases.*

**Supplementary Information:**

The online version contains supplementary material available at 10.1007/s00330-021-08385-9.

## Introduction

Neurological disorders represent a major public health problem in Europe and the rest of the world [1]. A systematic analysis for the Global Burden of Disease Study 2016 showed that neurological disorders were the leading cause of disability-adjusted life-years (worldwide 276 million) and the second leading cause of death (worldwide 90 million) [2].

Magnetic resonance imaging (MRI) technology and the development of MRI markers of neurological diseases have been improved substantially in both research and clinical environments over the last 30 years [3]. Automated MRI segmentation methods have been used in addition to visual analysis and manual segmentation assessments [4–6], improving the early diagnosis of neurological disease and the development of effective drugs [7–9]. In addition, large-scale multi-institutional research studies [10, 11] have worked in synergy for the implementation of standardized imaging acquisition protocols in the research environment and clinical setting [12, 13]. These advancements highlight a need for an MRI volumetric analysis tool suitable for routine clinical use. Few automated segmentation software are currently approved by regulatory agencies (such as the FDA) and therefore included in the clinical routine workflow.

To be validated and implemented in clinical practice, segmentation algorithms included in medical devices should demonstrate equal or better performance than the assessment performed by expert neuroradiologists. The present study aims to describe the comparison between the performance of the brain segmentation algorithms included in QyScore® and the manual segmentations or manual segmentation correction conducted by three expert neuroradiologists. Here we hypothesize that the segmentation algorithms included in QyScore® show reliable performance in comparison with the consensus of three expert neuroradiologists.

## Materials and methods

**Fig. 1:**
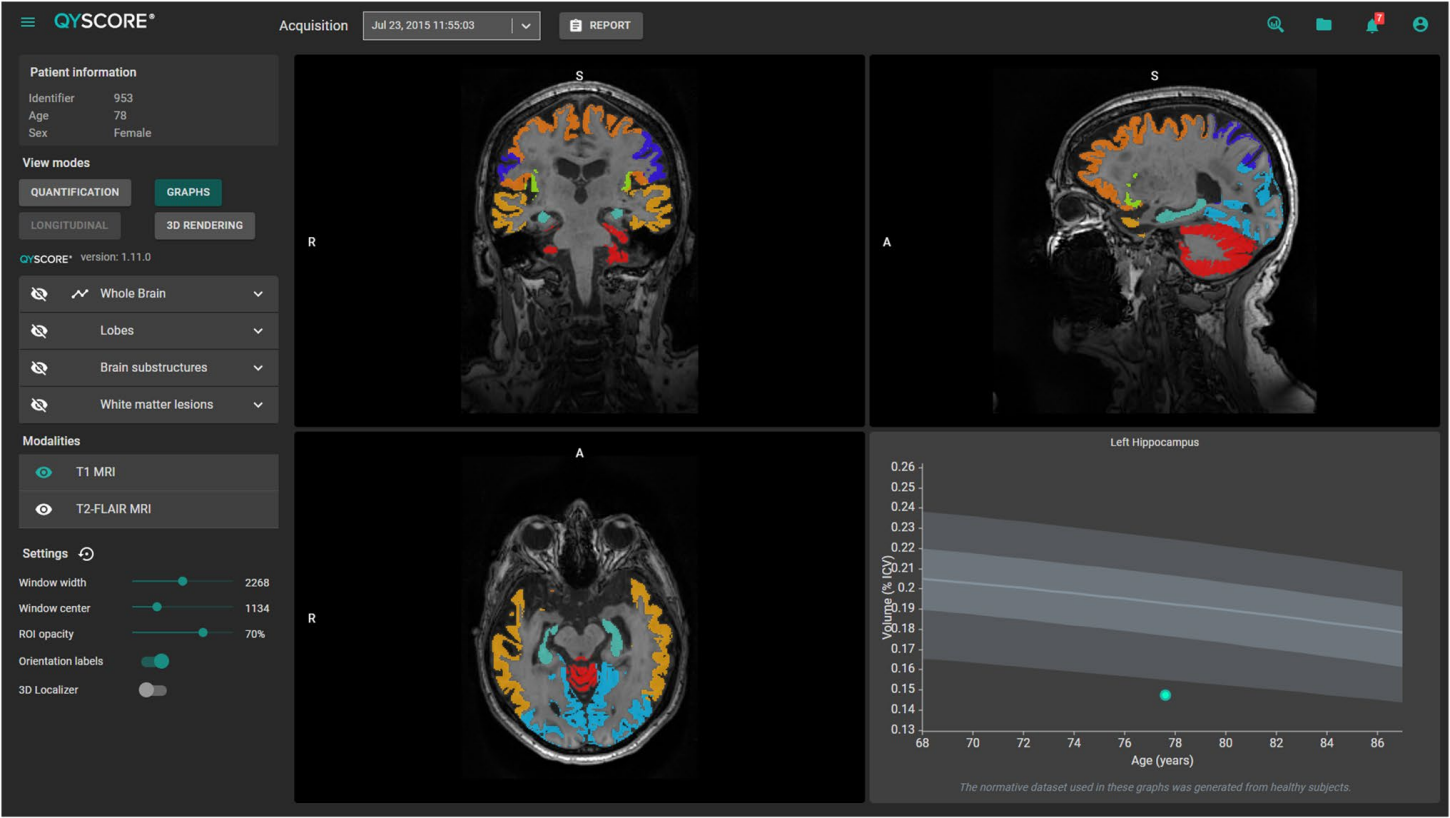
QyScore® visual interface, including 3D image navigation and volumetric results, compared with a normative dataset of healthy individuals

QyScore® is a CE-marked and FDA-cleared software, developed by Qynapse (https://www.qynapse.com/), that provides segmentation and volumetric measurements of grey matter (GM), white matter (WM), hippocampus (HP), and amygdala (AM) from 3DT1 images, as well as white matter hyperintensities (WMHs) from 3DT1 and FLAIR images. In addition, z-scores and percentiles are obtained from the comparison with a large normative database of healthy controls. The normative database includes cognitively intact individuals between the ages of 20 and 90 years, coming from European and North American databases [10, 11] (https://brain-development.org/ixi-dataset; https://www.humanconnectome.org/study/hcp-young-adult). The full panel of MRI markers described above is quantified in 15 min per patient. It has a user-friendly interface, including 3D navigation of MRI images (Fig. 1). The outputs of the software include an electronic report and color overlays of the regional segmentation on the selected brain image for visualisation.

### Populations and cohorts

The experimental validation of QyScore® was performed using data from different cohorts: the Alzheimer’s Disease Neuroimaging Initiative (ADNI) [10], the Open Access Series of Imaging Studies (OASIS) [14], the KIKI2009/Kirby [15], the Parkinson Progression Markers Initiative (PPMI) [11], the Frontotemporal Lobar Degeneration Neuroimaging Initiative (FTLDNI), the ANMerge dataset [16], and a public segmentation database of Multiple Sclerosis patients (LIMTS) [17]. Data from three additional cohorts were incorporated to enrich the sample: the Clinically Isolated Syndrome–COGnitive (SCI-COG) cohort, the REACTIV database, and the MEMORA cohort.

We created three different databases: one for the measurement of GM and WM volumes, one for HP and AM volumes, and one for WMH volumes. Subjects in each database were well-balanced according to age (ranging from 20 to 90 years old), sex, scanner field strength (1.5 T/3 T), and type of MRI acquisition (2D/3D for FLAIR images only). To ensure as broad a sample as possible in terms of volumetric measurements, we selected a heterogeneous population composed of healthy controls (HCs), mild cognitive impairment (MCI), Alzheimer’s disease (AD), Parkinson’s disease (PD), frontotemporal dementia (FTD), and multiple sclerosis (MS) patients.

The ADNI was launched in 2003 as a public–private partnership led by Principal Investigator Michael W. Weiner, MD. The primary goal of ADNI has been to test whether serial MRI, positron emission tomography (PET), biological markers, and clinical and neuropsychological assessment can be combined to measure the progression of MCI and AD. For up-to-date information, see http://adni.loni.usc.edu/www.adni-info.org.

The Open Access Series of Imaging Studies (OASIS) is a project aimed at making MRI data sets of the brain freely available to the scientific community. OASIS is made available by the Washington University Alzheimer’s Disease Research Center, Dr. Randy Buckner at the Howard Hughes Medical Institute (HHMI) at Harvard University, the Neuroinformatics Research Group (NRG) at Washington University School of Medicine, and the Biomedical Informatics Research Network (BIRN). It provides cross-sectional MRI data in young, middle aged, non-demented, and demented older adults [14].

FTLDNI was funded through the National Institute of Aging and started in 2010. The primary goals of FTLDNI were to identify neuroimaging modalities and methods of analysis for tracking frontotemporal lobar degeneration—and to assess the value of imaging versus other biomarkers in diagnostic roles. The principal investigator of FTLDNI was Dr. Howard Rosen, MD, at the University of California, San Francisco. The data are the result of collaborative efforts at three sites in North America. For up-to-date information on participation and protocol, please visit http://memory.ucsf.edu/research/studies/nifd

Clinical diagnostic criteria for each diagnosis considered were NINCDS/ADRDA and Clinical Dementia Rating Scale (CDR) = 1 for AD [14, 18]. PD was defined according to the features described by Marek and colleagues [11]. FTD was defined according to frontotemporal dementia consortium criteria [19] and MS, according to Polman and colleagues [20].

## MRI data

QyScore® analysis can be performed using MRI sequences with recommended acquisition parameters on 1.5- and 3-Tesla (1.5 T and 3 T) scanners, as reported in Table 1. More specifically, the software retrieves DICOM MRI data (non-contrast 3DT1 series acquired using 1.5 T or 3 T scanners and T2 fluid attenuated inversion recovery (FLAIR) series acquired using 3-Tesla scanners) from a DICOM server and sends them to an analysis server.

**Table 1:** Recommended neuroimaging parameters for analyzing QyScore® markers

	T1		T2FLAIR 2D		T2FLAIR 3D	
	Ideal	Tolerated	Ideal	Tolerated	Ideal	Tolerated
Voxel size in plane (mm × mm)	[1, 1]	Min: [0.43, 0.43] Max: [1.5, 1.5]	[1, 1]	Min: [0.43, 0.43] Max: [1.1, 1.1]	[1, 1]	Min: [0.43, 0.43] Max: [1.1, 1.1]
Field of view (mm)	*x*: [152, 340] *y*: [220, 340] *z*: [152, 340]	*x*: [144, 500] *y*: [170, 340] *z*: [132, 340]	*x*: [152, 340] *y*: [220, 340] *z*: [152, 340]	*x*: [144, 500] *y*: [170, 340] *z*: [132, 340]	*x*: [152, 340] *y*: [220, 340] *z*: [152, 340]	*x*: [144, 500] *y*: [170, 340] *z*: [132, 340]
Slice thickness (mm)	1	Min: 0.68 Max: 2	3.3	Min: 2 Max: 5	1	Min: 0.8 Max: 2
Interslice gap (mm)	0.0	Min: − 1.0 Max: 0	0.0	Min: 0.0 Max: 1.0	0.0	Min: − 1.0 Max: 0
Interpolation	No	Yes	No	Yes	No	Yes
Acquisition direction	Sagittal	Axial, Coronal	Axial	Coronal, Sagittal	Sagittal	Axial, Coronal
Acquisition type	3D	2D with isotropic voxel	2D	2D	3D	3D
Field strength	1.5 T, 3 T	1.5 T, 3 T	1.5 T, 3 T	1.5 T, 3 T	1.5 T, 3 T	1.5 T, 3 T

From the analysis server, before computing MRI analysis, QyScore® performs a quality check of MRI parameters to verify that the parameters are in line with the ones recommended (Table 1). The range of recommended parameters was selected for their suitability in a clinical routine setting. The analysis is performed if the parameters are within the “ideal” or “tolerated” ranges, as described in Table 1. When the acquisition parameters are not in line with the ones recommended, QyScore® does not perform the analysis. Then, QyScore® performs the automatic segmentation of GM, WM, HP, AM, and WMH.

For the present study, images were acquired using MRI scanners from different manufacturers: General Electric Healthcare (GE), Siemens Medical Solutions, Philips Medical Systems.

3D T1-weighted MRI images, used to quantify atrophy, were acquired either on 1.5 T or 3 T scanners, using exclusively gradient-echo 3D sequences. FLAIR images, used to quantify white matter hyperintensities, were acquired on 3 T scanners either in 2D or 3D.

## Automated Qyscore® imaging markers

All QyScore® imaging markers (GM, WM, HP, AM, and WMH volumes) are widely employed in the imaging field as markers of brain atrophy [21–24] and white matter hyperintensities [24]. The algorithms used for volume calculation are based on existing segmentation methods, as described in detail below. Fifteen minutes are needed for the segmentation of all structures (less than 10 min for T1-weighted MRI images only). Whole GM and WM volumes were quantified using the Statistical Parametric Mapping software v12 (SPM12). SPM is a software highly used for GM and WM segmentation both in research and in clinical data [25]. Hippocampus and AM volumes were measured using an improved version of SACHA (Segmentation Automatique Compétitive de l’Hippocampe et de l’Amygdale), a fast and fully automatic hybrid segmentation tool previously described in detail [26, 27].

White matter hyperintensity volumes were measured using a method based on the WHASA (White matter Hyperintensities Automated Segmentation Algorithm) automatic segmentation method [28].

## Expert consensus on manual segmentation and semi-automatic assessment of imaging markers

### Grey matter, white matter, hippocampus, and amygdala volumes

Three expert neuroradiologists manually edited and corrected the segmentation of GM and WM (rater’s initials: C.S., N.P., S.S.). First, GM and WM segmentations were performed using Freesurfer [29]. Then, GM and WM segmentations were corrected using the software ITK-SNAP according to their experience. Since no protocols were found in the literature, we asked clinicians to check the segmentation automatically performed to identify important segmentation errors, mainly at the cortex level.

Three expert neuroradiologists manually delineated HP and AM according to the anatomical constraints described by Chupin and colleagues [26].

For each marker considered (GM, WM, HP, and AM), we established a consensus from the corrections/segmentations obtained from the expert tracers by the Simultaneous Truth and Performance Level Estimation (STAPLE) algorithm. The STAPLE algorithm considers a collection of segmentations and computes a probabilistic estimate of the true segmentation by estimating an optimal combination of the segmentations [30]. The consensus was obtained using the Computational Radiology Kit (CRKIT) software that includes the STAPLE algorithm.

### White matter hyperintensity volume

Some of the data used for the validation of WHASA are publicly available (*N* = 30) [17]. We used the consensus derived from the WMH manual segmentation of three anonymous expert raters reported in the manuscript of Lesjak and colleagues [16]. On the remaining data (*N* = 100), segmentations were first initiated using the Lesion Segmentation Toolbox (LST) [31], then three expert neuroradiologists (rater’s initials: C.D., D.H., N.P.) manually corrected the WMH segmentation. The consensus among raters was obtained using the STAPLE algorithm.

## Measures of reliability

The reliability measures considered for the validation of QyScore® imaging markers were the dice similarity coefficient (DSC) and the relative volume difference (RVD) for GM, WM, HP and AM segmentations. The DSC, the absolute volume error (AVE), and the F1 metrics were considered for WMH segmentation. For each measure of reliability related to a QyScore® marker, we identified from the literature the performances of other segmentation methods comparable to QyScore®. As reported in Table 2 [26, 32–40], we averaged their performances to set fair values of reliability. We then considered these values of reliability as thresholds for the subsequent statistical analysis.

**Table 2:** Thresholds considered for each reliability measure

QyScore® markers	Thresholds			
	DSC	RVD (%)	AVE (mL)	F1
GM	0.8	9.59	–	–
WM	0.8	9.18	–	–
HP	0.7	31.5	–	–
AM	0.7	31.5	–	–
WMH low (< 5 mL)	0.27	–	2	–
WMH medium (5–15 mL)	0.51	–	5	–
WMH high (15–30 mL)	0.64	–	10	–
WMH very high (> 30 mL)	0.67	–	15	–
WMH whole sample	0.47	–	–	0.3

*Abbreviations*: *GM* grey matter, *WM* white matter, *HP* hippocampus, *AM* amygdala, *WMH* white matter hyperintensity, *DSC* dice similarity coefficient, *RVD* relative volume difference, *AVE* absolute volume error, *SD* standard deviation. Values in parentheses indicate references

### Dice similarity coefficient

The DSC [35] is the most frequently used statistical validation metric employed to evaluate the performance of both the reproducibility of manual segmentations/corrections and the spatial overlap accuracy of automated segmentation methods [4]. We identified from the literature DSC thresholds for each QyScore® marker to test our statistical hypothesis as reported in Table 2. The values identified in Table 2 are usually considered an excellent match between two segmentations and are in line with the values reported in previous studies [32]. The DSC threshold for WMH segmentation was defined based on four categories of lesions load: low (< 5 mL), medium (5–15 mL), high (15–30 mL), very high (> 30) mL). The DSC thresholds for these four categories of WMH were obtained by averaging the DSC values reported in the supplementary material of the study by Commowick [34] et al. for each category considered. Categories and DSC thresholds are described in Table 2.

### The relative volume difference

We calculated the RVD between the volume of the structure automatically segmented and the volume obtained from the consensus of the three expert neuroradiologists. The RVD threshold (Table 2) was obtained among the different RVD values acquired from freeware packages widely used by experts and described in the manuscript of Mendrik and colleagues [41]. To the best of our knowledge, this study is the only one reporting several RVD values for GM and WM segmentation [41]. RVD thresholds for the HP and AM, reported in Table 2, represent the average of RVDs from a selection of studies published in the literature [36, 37, 42–46].

### The absolute volume error and the F1 score

We measured the AVE and F1 score exclusively for WMH. Evaluation of WMH detection relies on determining how many WMH have been correctly or incorrectly detected. The F1-score ranges from 0 to 1 and provides an idea of the detection performance (perfect detection: 1). It is calculated from (i) the number of WMH in each segmentation (expert consensus and QyScore® automatic segmentation), (ii) the number of WMH correctly detected from the experts’ consensus, and (iii) the number of WMH in the automatic segmentation for which there is a WMH from the consensus, as suggested by Commowick and colleagues [34].

Thresholds for both metrics (AVE and F1 score) for WMH were defined using the values reported in the supplementary material of the study conducted by Commowick and colleagues [34] (Table 2).

## Statistical analysis

The validity of QyScore® imaging markers was tested in comparison to the consensus obtained by three experts. We identified thresholds of reliability measures based on the performances reported in the literature from other similar segmentation methods (Table 2). The null hypothesis was that the measures of comparison DSC/F1 scores (the higher, the better) were equal to/below the chosen threshold—and the alternative hypothesis was that the metrics were higher, indicating improved accuracy when comparing QyScore® with the consensus obtained by three experts. For DSC and F1 scores, the lower bound of the 97.5% confidence interval was compared to the threshold, indicating that the DSC and F1 scores obtained using QyScore® were significantly better to the DSC and F1 scores reported in the literature for similar segmentation methods. For RVD and AVE (the lower, the better), the null hypothesis was that the metrics were equal to/above the threshold, and the alternative hypothesis is that the metrics were lower. The upper bound of the 97.5% confidence interval was compared to the threshold, indicating that the RVD and AVE obtained using QyScore® were significantly inferior to the RVD and AVE reported in the literature for similar segmentation methods.

Furthermore, we compared volumes obtained from the automated QyScore® imaging markers with the consensus obtained by three experts using regression analysis and Bland-Altmann plots.

We used the following library in Python to perform statistical analysis: Scikit-learn (http://scikit-learn.org/stable/), version 0.19.1; Scipy (https://www.scipy.org/), version 0.17.0; NumPy (http://www.numpy.org/), version 1.14.3; Nipype (https://nipype.readthedocs.io), version 1.1.2.

## Results

All 180 MRI images passed the quality control performed by QyScore®.

Table 3 describes the main features of each database’s selection criteria and the mean absolute volume of each QyScore® marker. Each database was furthermore constituted of HC and clinical patients as follows: GM and WM database (24 HC, 2 AD, 2 MS, 2 PD), HP and AM database (37 HC, 4 AD, 6 MS, 3 PD), WMH database (20 HC, 49 AD, 6 FTD, 45 MS, 2 PD, 10 with MCI). The type of manufacturers used for the acquisition of the MRI images was distributed among GM and WM database (2 GE, 9 Philips, 19 Siemens), HP and AM database (4 GE, 13 Philips, 33 Siemens), WMH database (30 GE, 26 Philips, 74 Siemens). Consensus results and the contribution of each expert neuroradiologist EW reported in Table 4.

**Table 3:** Distribution of selection criteria (sex, age, magnetic field, type) in each database and absolute volumes of QyScore® markers

Database		Volumes (mL) (mean (sd))	Sex (M/W)	Age mean (sd)	Magnetic field (1.5 T/3 T)	Type (2D/3D)
GM and WM (*N* = 30)	GM	672.67 (82.02)	16/14	53.51 (20.48)	15/15	0/30
	WM	462.19 (51.85)				
HP and AM (*N* = 50)*	HP	5.83 (0.71)	27/23	52.84 (20.40)	24/26	0/50
	AM	2.90 (0.43)				
WMH (*N* = 130)		18,76 (17,62)	60/70	62.95 (19.35)	0/130	70/60

*Abbreviations*: *M* men, *W* women, *GM* grey matter, *WM* white matter, *HP* hippocampus, *AM* amygdala, *WMH* white matter hyperintensity, *sd* standard deviation

*The 30 images included for the GM and WM analysis were also included in the dataset for the HP and AM analysis

**Table 4:** Average measures and standard deviations of overlap and volumetric agreement between the segmentation performed by each neuroradiologist and their consensus obtained using the STAPLE algorithm for each QyScore® marker

		DICE	RVD	AVE	F1
GM (*N* = 30)	Manual tracer 1	0.99 (0.01)	0.18 (0.33)	–	–
	Manual tracer 2	0.99 (0.01)	0.08 (0.06)	–	–
	Manual tracer 3	1.00 (0.01)	0.04 (0.04)	–	–
WM (*N* = 30)	Manual tracer 1	0.99 (0.01)	0.15 (0.43)	–	–
	Manual tracer 2	0.99 (0.08)	0.09 (0.08)	–	–
	Manual tracer 3	0.99 (0.01)	0.07 (0.07)	–	–
HP (*N* = 50)	Manual tracer 1	0.98 (0.07)	1.38 (2.00)	–	–
	Manual tracer 2	0.97 (0.01)	2.67 (3.24)	–	–
	Manual tracer 3	0.95 (0.01)	3.85 (2.27)	–	–
AM (*N* = 50)	Manual tracer 1	0.90 (0.11)	7.91 (10.59)	–	–
	Manual tracer 2	0.86 (0.11)	12.59 (12.78)	–	–
	Manual tracer 3	0.89 (0.11)	5.56 (6.94)	–	–
WMH (*N* = 100)	Manual tracer 1	0.88 (0.12)	–	2.70 (4.75)	0.83 (0.16)
	Manual tracer 2	0.85 (0.18)	–	2.51 (3.21)	0.70 (0.23)
	Manual tracer 3	0.77 (0.21)	–	1.50 (1.80)	0.62 (0.27)

*Abbreviations*: *GM* grey matter, *WM* white matter, *HP* hippocampus, *AM* amygdala, *WMH* white matter hyperintensity, *DSC* dice similarity coefficient, *RVD* relative volume difference, *AVE* absolute volume error; values in parentheses indicate standard deviation

### Validation results for GM and WM segmentations

**Fig. 2:**
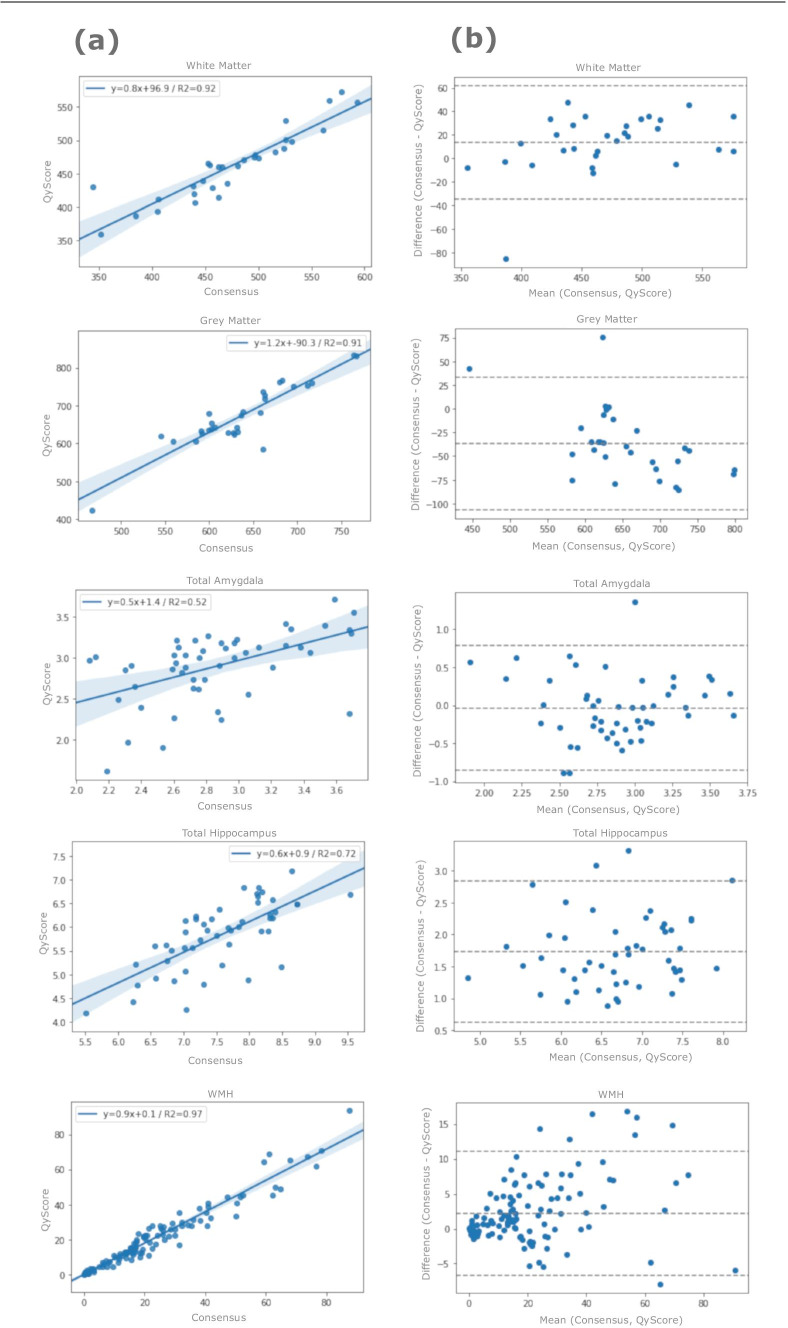
Scatterplots from regression analysis (**a**) and Bland–Altman plots (**b**) showing good concordance between QyScore® markers and the consensus from the expert raters

Comparison with the experts’ consensus showed that GM and WM segmentations, obtained using QyScore®, displayed the lower bound of the DSC confidence intervals (GM 97.5% CI: 0.848, 0.866; WM 97.5% CI: 0.892, 0.907 respectively) above the DSC threshold (0.8), as well as the higher bounds of the RVD confidence interval (GM 97.5% CI: 5.578, 8.464; WM 97.5% CI: 2.985, 6.425) below the RVD threshold (9.59% for the GM and 9.18% for the WM respectively). We found consistent results after the stratification by field strength; the mean DSC for the GM was equal to 0.87 for 1.5 T and 0.86 for 3 T; the mean DSC for the WM was equal to 0.92 for 1.5 T and 0.90 for 3 T. Coefficient of determination was equal to 0.91 for the GM and 0.92 for the WM (Fig. 2A), whilst the means (95% confidence interval (CI)) of Bland–Altman plots were − 36.51 (95% CI: 35.39, − 108.42) for GM and 13.33 (95% CI: 63.04, − 36.38) for WM (Fig. 2B).

### Validation results for HP and AM segmentations

HP and AM segmentations showed the lower bound of the DSC confidence intervals (HP 97.5% CI: 0.801, 0.818; AM 97.5% CI: 0.763, 0.786 respectively) above the DSC threshold (0.7), as well as the higher bounds of the RVD confidence interval (HP 97.5% CI: 20.886, 24.648; AM 97.5% CI: 9.203, 15.023) below the RVD threshold (31.5%). We found consistent results after the stratification by field strength; the mean DSC for the HP was equal to 0.81 for 1.5 T and 0.80 for 3 T; the mean DSC for the AM was equal to 0.75 for 1.5 T and 0.76 for 3 T.

Coefficient of determination was equal to 0.72 for the HP and 0.52 for the AM (Fig. 2A), while the means (95% CI) of Bland–Altman plots were − 1.63 (95% CI: − 0.93, − 3.23) for HP and 0.02 (95% CI: 0.86, − 0.81) for AM (Fig. 2B).

### Validation results for WMH segmentations

As with the previous markers, WMH segmentations also satisfied the alternative hypothesis for each comparison considered. As reported in Table 5, for all the WMH load considered, the lower bound of the DSC confidence intervals was above the DSC threshold, and the higher bound of the AVE confidence interval was below the AVE threshold.

**Table 5:** Thresholds of the reliability measures (DSC, AVE, F1) for each category of WMH as well as confidence intervals of WMH volumes measured using QyScore® in comparison with experts’ consensus

QyScore® markers	Thresholds			QyScore® 97.5% confidence intervals		
	DSC	AVE (mL)	F1	DSC	AVE (mL)	F1
WMH low (< 5 mL)	0.27	2	–	**0.284**, 0.414	0.329, **0.666**	–
WMH medium (5–15 mL)	0.51	5	–	**0.647**, 0.716	1.152, **2.518**	–
WMH high (15–30 mL)	0.64	10	–	**0.728**, 0.771	2.648, **4.120** Ok	–
WMH very high (> 30 mL)	0.67	15	–	**0.761**, 0.811	6.186, **10.417**	–
WMH whole sample	0.47	–	0.3	**0.616**, 0.674	–	**0.352**, 0.3911

Bold numbers highlight the fact that the values were above or below the defined tresholds

*Abbreviations*: *WMH* white matter hyperintensity, *DSC* dice similarity coefficient, *AVE* absolute volume error, *mL* milliliter

Coefficient of determination was equal to 0.97 (Fig. 2A), while the mean (95% CI) of Bland–Altman plot was − 2.16 (95% CI: 11.17, − 6.84) (Fig. 2B).

## Discussion

Our results showed that the fully automatic tool, QyScore®, accurately measures brain imaging markers such as GM, WM, HP, AM, and WMH volumes. In comparison with the consensus of three expert neuroradiologists, QyScore® showed good concordance for all its imaging markers: GM, WM, HP, AM, and WMH—as depicted by the Bland–Altman plots and similarity measures. These results support the reliability of QyScore® compared with the experts’ consensus in segmenting brain structure essentials for detecting brain atrophy in several brain diseases such as Alzheimer’s, Parkinson’s, and multiple sclerosis. The value of reliability indices (DSC, AVE, RVD, F1), obtained from the comparison between QyScore® and the consensus from three expert neuroradiologists, satisfied the alternative hypothesis for each comparison considered. Thresholds were set based on data present in the literature using other automatic segmentation methods, suggesting that QyScore® performs equally to or better than other automated methods currently used in the research context. In addition, results obtained by QyScore® are consistent using T1-weighted and FLAIR sequences from common clinical platforms and regardless of field strength for T1-weighted images.

### Grey matter and white matter

The performance of markers quantified using QyScore® is in accordance with the currently available methods for the automatic segmentation of brain MRI described in the literature.

In particular, results from the MRBrainS challenge—comparing automatic and semi-automatic methods to segment GM and WM to the gold standard—concluded that SPM12 was the most robust, accurate, and fastest algorithm among the freeware packages evaluated [41]. A further study assessing whole brain and GM atrophy in multiple sclerosis showed for GM a DSC equal to 0.90 for SPM12 that was significantly higher to the one found for MSmetrix (DSC = 0.59) [47]. The quantification of GM atrophy using QyScore® in a heterogeneous sample of individuals with different pathophysiology showed an intermediate DSC (GM 97.5% CI: 0.848–0.866).

### Hippocampus and amygdala

Regarding the HP and AM segmentation, a previous version of QyScore®’s algorithm using SACHA has shown better segmentation results compared to semi-automatic methods and other segmentation methods based on atlases [48]. The overlap between SACHA segmentation and the manual segmentation (detected by the DSC) was higher compared to the ones obtained for Freesurfer and FSL/FIRST [33]. HP measurements with Freesurfer were superior to FIRST [33]; however, several studies reported an overestimation of HP volume measured using Freesurfer [33, 46, 49]. Moreover, SACHA showed good accuracy in detecting mild cognitive impairment (MCI) and AD patients [50].

### White matter hyperintensities

A previous version of QyScore®’s algorithm WHASA was validated on clinical routine MRI images showing a high intra-class coefficient of correlation with manual segmentation [28], which supports our results. Furthermore, WHASA was previously compared to other methods such as Freesurfer, a thresholding approach, and other methods based on k-nearest neighbors (kNN) and support vector machine (SVM) algorithms [28]. WHASA showed a better performance than Freesurfer and the thresholding approach as well as a comparable performance to the one obtained from kNN and SVM methods [28]. The spatial overlap between WHASA segmentation and manual segmentation was higher compared to the spatial overlap that was found between established automatic methods and manual segmentation [51].

### Implications of the use of automatic algorithms and medical devices in clinical routine

Several medical devices measuring brain imaging markers for neurological diseases are currently available [51–55]. However, multiple elements prevent the comparison between QyScore® performances and other devices due to (i) different indices of reliability used for their validation [54], (ii) the use of different validation methods [53, 56], and (iii) heterogeneous target populations among studies (exclusively young healthy controls, or MS or AD patients only) [51, 53, 54].

Volumetric MRI measures (such as the HP or GM volume) have been proposed as surrogate markers of in vivo brain atrophy and neurodegeneration [57, 58] and might be used for the early diagnosis, monitoring, and secondary outcome in clinical trials for several neurological diseases. An additional advantage of measuring brain atrophy in clinical trials is that fewer subjects need to be included [59, 60].

In line with our evidence, a recent research study has also demonstrated that quantitative reports, alongside routine visual MRI assessment, improves sensitivity and accuracy for detecting volume loss in AD compared to visual assessment alone [61].

Gold standard methods, such as manual tracing, are difficult, time-consuming and prone to inter- and intra-rater variability. In contrast, automatic methods have the obvious advantage of being consistent and fast compared to manual or semi-automatic methods [62]. For this reason, the validation of automated measures of MRI brain segmentation is of substantial importance to support practitioners in clinical settings and pharmacological companies in the context of new drug development [46].

Thanks to the reliability of the main results presented in the present manuscript, QyScore® has been considered suitable by regulatory bodies worldwide (CE and FDA). The panel of MRI markers (GM, WM, HP, AM, and WMH volumes) measured by QyScore® can support clinicians in the diagnosis and monitoring of clinical progression of neurological diseases such as MCI and AD dementia, MS, Parkinson’s, and other neurodegenerative disorders. However, the clinicians make the final clinical decision based on their expert review of the QyScore® results.

The automated measure of MRI markers allowing fast segmentation of brain volumes and WMH (15 min) overcomes the time-consuming and subjective nature of the manual approach. QyScore® has reliable markers that can be implemented in clinical trials as primary/secondary outcomes to investigate the disease-modifying effect of treatments. In this regard, the HP atrophy measured using SACHA was already employed as the primary endpoint in a clinical trial aimed at investigating the efficacy of donepezil treatment in suspected prodromal AD patients [9].

Furthermore, QyScore® results can be incorporated into the clinical reports—providing additional neuroimaging information to clinicians that may be employed during their clinical and radiological assessments. QyScore® outputs can assist clinicians in the clinical diagnosis and monitoring, as well as in the choice of the most appropriate treatment in clinical practice.

### Study limitations

Some limitations of the present study should be considered. The current version of the QyScore® algorithm for GM segmentation is not optimized for the exclusion of white matter lesions. In this regard, review of results is required before considering their use for clinical reports. This functionality will be included in a future update of the software. QyScore® algorithms have been mainly validated on open-source research cohorts, and further studies are ongoing in a clinical routine setting. A direct comparison with other medical devices, test–retest, and longitudinal data is also needed.

## Conclusions

QyScore® provides reliable automatic segmentation of brain structures compared to the experts’ consensus and other semi-automatic and automatic software described in the literature. Our results support the implementation of medical devices, such as QyScore® using neuroimaging methods, in clinical routine for supporting the diagnosis and monitoring of brain disorders.

QyScore® markers could also be implemented in clinical trials to test the efficacy of new drugs for neurological diseases. Reliable measures of brain atrophy and white matter hyperintensities, such as the ones provided by QyScore®, will help us move into more personalized and evidence-based medicine for neurological diseases. Further retrospective and prospective validation studies, as well as test–retest reliability studies, are currently ongoing on real-world data to demonstrate the diagnostic performance and the clinical impact of using QyScore® in neurological disorders.

## Supplementary Information

Below is the link to the electronic supplementary material.

Supplementary file1 (XLSX 28 KB)

## Abbreviations

AD: Alzheimer’s disease
ADNI: Alzheimer’s Disease Neuroimaging Initiative
AM: Amygdala
AVE: Absolute volume error
DSC: Dice similarity coefficient
FDA: Food and Drug Administration
FLAIR: T2 fluid attenuated inversion recovery
FTD: Frontotemporal dementia
FTLDNI: Frontotemporal Lobar Degeneration Neuroimaging Initiative
GM: Grey matter
HC: Healthy controls
HP: Hippocampus
kNN: K-nearest neighbors
MC: Multiple sclerosis
MCI: Mild cognitive impairment
MRI: Magnetic resonance imaging
NINCDS/ADRD: National Institute of Neurological and Communicative Disorders and Stroke and the Alzheimer’s Disease and Related Disorders Association
OASIS: Open Access Series of Imaging Studies
PD: Parkinson’s disease
PPMI: Parkinson Progression Markers Initiative
RVD: Relative volume difference
SPM12: Statistical Parametric Mapping software v12
SVM: Support vector machines
WM: White matter
WMH: White matter hyperintensity

## Declarations

### Guarantor

The scientific guarantor of this publication is Enrica Cavedo.

### Statistics and biometry

One of the authors has significant statistical expertise.

### Study subjects or cohorts overlap

Some study subjects or cohorts have been previously reported in previous studies for the all the public available cohorts cited in the Manuscript.

### Methodology

• retrospective

• cross-sectional study

• multicenter study
